# Is isolated aortic valve replacement sufficient to treat concomitant moderate functional mitral regurgitation? A propensity-matched analysis

**DOI:** 10.1186/s13019-018-0760-3

**Published:** 2018-06-19

**Authors:** Robert A. Sorabella, Anna Olds, Halit Yerebakan, Dua Hassan, Michael A. Borger, Michael Argenziano, Craig R. Smith, Isaac George

**Affiliations:** Division of Cardiothoracic Surgery, New York Presbyterian Hospital - Columbia University College of Physicians and Surgeons, 177 Fort Washington Ave, MHB 7GN-435, New York, NY 10032 USA

**Keywords:** Aortic valve replacement, Mitral valve disease, Mitral regurgitation

## Abstract

**Background:**

A significant proportion of patients presenting for isolated aortic valve replacement (AVR) demonstrate some degree of functional mitral regurgitation (fMR). Guidelines addressing concomitant mitral valve intervention in those patients with moderate fMR lack strong evidence-based support. Our aim is to determine the effect of untreated moderate fMR at the time of AVR on long-term survival.

**Methods:**

All patients undergoing isolated AVR from 2000 to 2013 at our institution were retrospectively reviewed. Patients were stratified according to severity of preoperative fMR; 0–1+ MR (Group NoMR, *n* = 1826) and 2–3+ MR (Group MR, *n* = 330). All patients in Group MR were propensity-matched with patients in Group NoMR to control for differences in baseline characteristics. The primary outcome of interest was overall survival.

**Results:**

Propensity analysis matched 330 patients from each group. Mean age was 77.9 ± 10.0 years and 50.6% were male. There were no differences in baseline demographics, echocardiographic parameters, or co-morbidities between groups. Kaplan-Meier analysis showed significantly worse medium and long-term survival in Group MR compared to Group NoMR (log-rank *p* = 0.02). Follow-up echocardiography showed slightly more severe MR in Group MR (1.1 ± 0.7 MR vs. 0.8 ± 0.7 NoMR, *p* = 0.03) at 1 year.

**Conclusions:**

Patients undergoing isolated AVR with concomitant 2–3+ fMR experience poorer long-term survival than those patients with no or mild fMR. This suggests that mitral valve intervention may be necessary in patients undergoing AVR with clinically significant fMR.

## Background

Surgical aortic valve replacement (AVR) remains the most common valvular operation with over 50,000 procedures performed in the United States in 2013 alone [1]. In addition to correction of primary aortic valve pathology, current American Heart Association guidelines recommend mitral valve intervention for patients presenting with concomitant severe functional mitral regurgitation (fMR) as a result of their aortic valve disease [2–4]. However, according to published literature, up to two-thirds of patients with aortic stenosis or insufficiency (AS, AI, respectively) can present with moderate or less fMR, and significant debate persists on whether these patients warrant mitral valve repair at the time of AVR [5–12].

The traditional, conservative perspective assumes that non-severe fMR regresses following isolated AVR due to left ventricular reverse remodeling and removal of afterload obstruction, which subsequently leads to improved mitral leaflet coaptation. Although this phenomenon occurs to some extent following AVR, some reports suggest that patients may still be left with clinically significant fMR even at late follow-up, which may lead to worsened long-term survival [13–15]. However, these studies are limited by small sample sizes and lack of extended follow-up, and fail to yield definitive conclusions. Therefore, the aim of this study is to determine the impact of uncorrected moderate fMR at the time of AVR on late survival in comparison to patients with no or mild fMR in a large patient population. These results may have important clinical implications in defining the appropriate treatment strategy for patients with combined aortic and moderate functional mitral valve disease, particularly in the modern era of transcatheter valve therapy.

## Methods

### Patient selection

All patients undergoing isolated AVR at NY Presbyterian-Columbia University Medical Center between January 2000 and December 2013 were retrospectively reviewed for inclusion into the study. Patients with severe fMR (4+), primary mitral valve disease, prior mitral surgery, or cardiogenic shock at the time of surgery were excluded from the analysis. A total of 2156 patients met inclusion and exclusion criteria, and patients were stratified according to degree of preoperative fMR: 0–1+ MR (Group NoMR, *n* = 1826) and 2–3+ MR (MR, *n* = 330). Functional MR was defined as MR with normal mitral valve morphology regardless of severity of left ventricular dysfunction as evaluated by preoperative transthoracic or transesophageal echocardiography. Degree of MR was assessed using a 0–4+ scale, as graded by a blinded echocardiographer (0 = no MR, 1 + =mild MR, 2 + =moderate MR, 3 + =moderate-severe MR, 4 + =severe MR). Given the significant underlying differences in demographics and co-morbid conditions between groups, a propensity-matched analysis was performed using a subset of the overall cohort. The study was approved by the Columbia University Institutional Review Board and need for individual patient consent was waived.

Clinical and follow-up data were collected from the electronic medical record and mortality data for patients lost to follow up were collected from United States Social Security Death Index. Baseline demographics, co-morbidities [congestive heart failure, prior myocardial infarct, severe chronic kidney disease (eGFR < 30 mL/min), diabetes mellitus, end-stage renal disease needing dialysis, cerebrovascular disease, peripheral vascular disease, and chronic obstructive pulmonary disease], preoperative echocardiographic measurements, operative details, postoperative complications and length of stay, follow-up echocardiographic data, and survival data were collected for analysis.

### Statistical analysis

All analyses were conducted using SPSS version 22 (IBM corporation, Armonk, NY). Continuous variables are presented as mean ± standard deviation and compared using independent samples t-tests, or median and interquartile range and compared using Mann-Whitney U test where appropriate. Categorical variables are presented as total count and percentage of the group, and compared using Pearson’s chi-square test or Fisher’s exact test where applicable. Kaplan-Meier analysis was used for comparison of survival, and survival curves were compared using the log-rank test. In order to control for differences in preoperative variables between groups, a propensity-matched analysis was performed. Patients were assigned a propensity score and matched using the nearest neighbor Greedy 5 to 1 digit matching algorithm (MatchIt package in R 3.0.2, R foundation for Statistical Computing, Vienna, Austria). Covariates included in calculation of the propensity score included age at surgery, gender, body mass index, preoperative ejection fraction, preoperative tricuspid regurgitation, preoperative hemoglobin, indication for surgery, and history of severe chronic kidney disease, cerebrovascular disease, myocardial infarction, peripheral vascular disease, chronic obstructive pulmonary disease, diabetes mellitus, need for hemodialysis, congestive heart failure, or prior cardiac surgery. Matching was done in a 1:1 fashion and matched 330 patients from each group for comparison. All *p*-values≤0.05 were considered statistically significant.

## Results

### Overall cohort analysis

Baseline characteristics, operative details, and outcomes of the overall cohort analysis are presented in Table 1. Patients in Group MR were significantly older, had lower BMIs, and generally had more preoperative co-morbidities than those in Group NoMR. Preoperative echocardiograms revealed significantly lower left ventricular ejection fractions and higher rates of severe aortic stenosis (AS) and severe tricuspid regurgitation (TR) in Group MR. Analysis of postoperative complications showed that patients in Group MR had a significantly higher 30-day mortality rate (3.6% MR vs. 1.5% NoMR, *p* = 0.007) and experienced significantly longer postoperative lengths of stay and higher rates of postoperative respiratory failure.

**Table 1:** Overall Cohort Analysis

	NoMR	MR	*p*-value
*Demographics*			
Total, n	1826	330	–
Age, years (mean ± SD)	69.3 ± 14.5	78.1 ± 10.1	< 0.001
Male, n (%)	1061 (58.1)	174 (52.7)	0.07
BMI, kg/m^2^ (mean ± SD)	28.0 ± 5.8	27.2 ± 5.6	0.03
*Co-morbidities, n (%)*			
Myocardial infarction	156 (8.5)	66 (20.0)	< 0.001
Congestive heart failure	329 (18.0)	111 (33.6)	< 0.001
Severe CKD	81 (4.4)	39 (11.8)	< 0.001
Diabetes	361 (19.8)	84 (25.5)	0.02
Dialysis	26 (1.4)	9 (2.7)	0.09
*Baseline echocardiography*			
LVEF, % (mean ± SD)	52.1 ± 12.0	46.1 ± 14.2	< 0.001
3–4+ AI, n (%)	424 (23.2)	65 (19.7)	0.16
Severe AS, n (%)	1472 (80.6)	287 (87.0)	0.006
3–4+ TR, n (%)	16 (0.9)	11 (3.3)	< 0.001
*Operative details*			
Re-operations, n (%)	324 (17.7)	96 (29.1)	< 0.001
CPB time, minutes (mean ± SD)	88.9 ± 32.0	92.5 ± 33.2	0.06
XCL time, minutes (mean ± SD)	62.3 ± 19.8	63.3 ± 18.8	0.39
*Outcomes*			
30-day mortality, n (%)	27 (1.5)	12 (3.6)	0.007
Post-op LOS, days (median, IQR)	7, 5–9	8, 6–11	< 0.001
Re-op for bleeding	60 (3.3)	12 (3.6)	0.74
Respiratory failure, n (%)	91 (5.0)	26 (7.9)	0.03
New need for dialysis, n (%)	18 (1.0)	3 (0.9)	0.90
*Echocardiographic follow-up*			
1-year MR grade (mean ± SD)	0.6 ± 0.7	1.1 ± 0.7	< 0.001

Abbreviations: AI = aortic insufficiency, AS = aortic stenosis, BMI = body mass index, CKD = chronic kidney disease, CPB = cardiopulmonary bypass, IQR = interquartile range, LOS = length of stay, LVEF = left ventricular ejection fraction, MR = mitral regurgitation, TR = tricuspid regurgitation, XCL = aortic cross-clamp

**Fig. 1:**
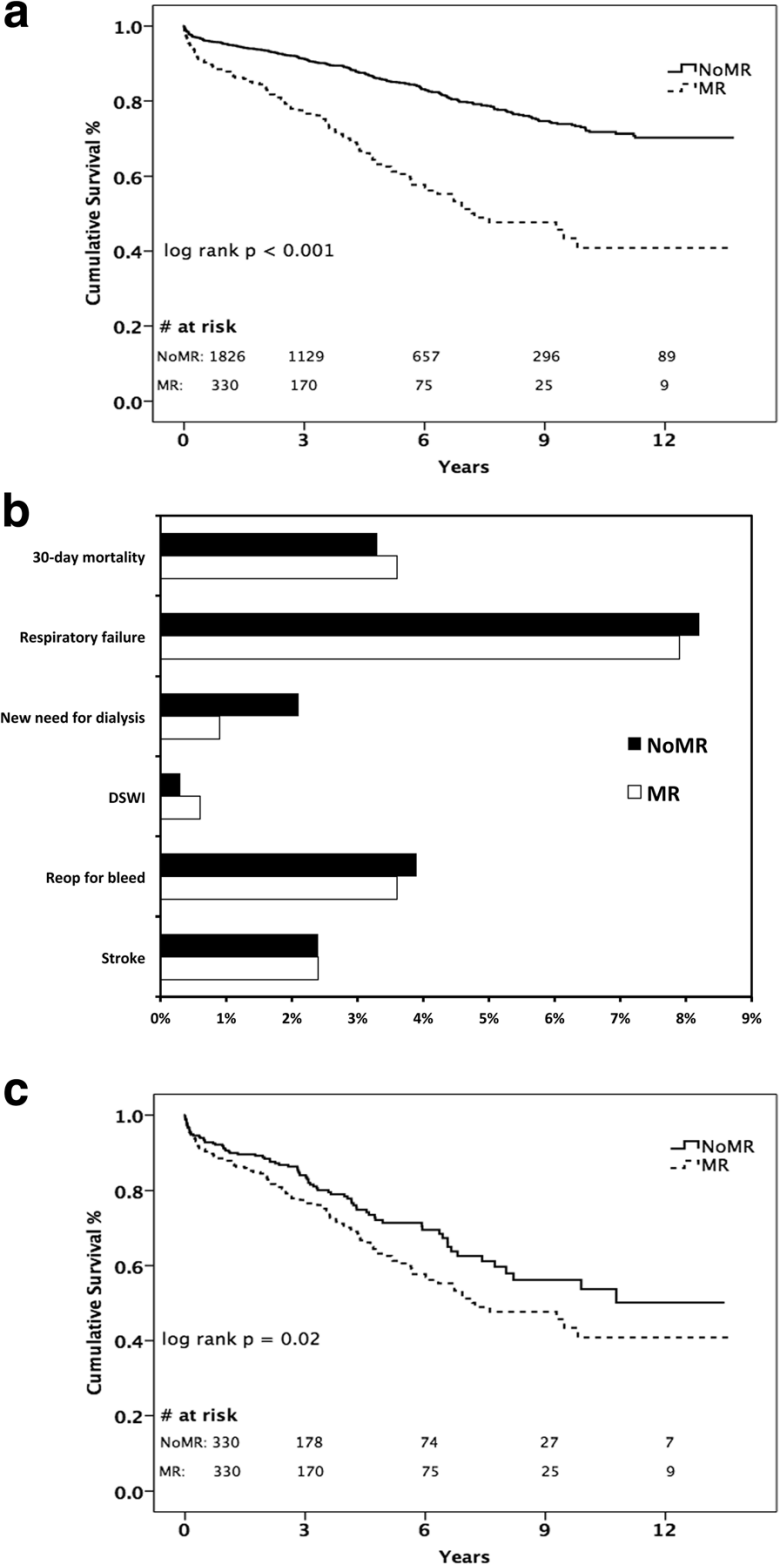
**a** Kaplan-Meier analysis of long-term survival in overall cohort stratified by treatment group, (**b**) Postoperative complications of propensity-matched cohort by treatment group, (**c**) Kaplan-Meier analysis of long-term survival in propensity-matched cohort by treatment group. (Abbreviations: DSWI = deep sternal wound infection)

Kaplan-Meier analysis of survival in the overall cohort (Fig. 1A) showed significantly worse medium- and long-term survival for patients in Group MR (log rank *p* < 0.001). In addition, follow-up echocardiograms showed more severe MR at 1 year in Group MR (1.1 ± 0.7 MR vs. 0.6 ± 0.7 NoMR, *p* < 0.001) compared to Group NoMR, although the mean severity of MR in both groups fell in the trace-to-mild range.

### Propensity-matched analysis

In view of the significant baseline differences between the groups MR and NoMR, a propensity-matched analysis was performed. A total of 330 patients were identified in each group following propensity matching. Baseline characteristics are shown in Table 2. Mean age was 77.9 ± 10.0 years and 50.6% of patients were male. There were no significant differences in co-morbidities. Propensity-matched preoperative echocardiographic data is presented in Table 3. There was no difference in preoperative ejection fraction or prevalence of severe AI, severe AS, or severe TR. Propensity-matched operative details are presented in Table 4. There was no difference in patients undergoing re-operative sternotomy or a minimally invasive approach. The vast majority of patients (*n* = 298 (90.3%) in MR vs. *n* = 299 (90.6%) in NoMR, *p* = 0.9) underwent bioprosthetic AVR with no differences between groups.

**Table 2:** Propensity-Matched Baseline Characteristics

	NoMR	MR	*p*-value
*Demographics*			
Total, n	330	330	–
Age, years (mean ± SD)	77.7 ± 10.0	78.1 ± 10.1	0.66
Male, n (%)	160 (48.5)	174 (52.7)	0.28
BMI, kg/m^2^ (mean ± SD)	27.2 ± 5.8	27.2 ± 5.7	0.99
*Co-morbidities, n (%)*			
Myocardial infarction	64 (19.4)	66 (20.2)	0.85
Congestive heart failure	111 (33.6)	111 (33.6)	1.00
Cerebrovascular disease	33 (10.0)	43 (13.0)	0.22
Severe CKD	33 (10.0)	39 (11.8)	0.45
Peripheral vascular disease	31 (9.4)	31 (9.4)	1.00
COPD	46 (13.9)	45 (13.6)	0.91
Diabetes	84 (25.5)	66 (25.5)	1.00
Dialysis	9 (2.7)	9 (2.7)	1.00

Abbreviations: BMI = body mass index, CKD = chronic kidney disease, COPD = chronic obstructive pulmonary disease

**Table 3:** Propensity-Matched Baseline Echocardiographic Data

	NoMR	MR	*p*-value
Ejection fraction			
LVEF, % (mean ± SD)	46.7 ± 14.7	46.1 ± 14.2	0.61
LVEF > 50%, n (%)	162 (50.9)	158 (47.9)	0.76
LVEF 30–50%, n (%)	124 (37.6)	128 (38.8)	0.75
LVEF < 30%, n (%)	44 (13.3)	44 (13.3)	1.00
Aortic insufficiency, n (%)			
None/Trace (0)	136 (41.2)	128 (38.8)	0.52
Mild (1+)	92 (27.9)	76 (23.0)	0.15
Moderate (2+)	47 (14.2)	61 (18.5)	0.14
Moderately-Severe/Severe (3–4+)	55 (16.7)	65 (19.7)	0.31
Aortic stenosis, n (%)			
None	33 (10.0)	34 (10.3)	0.90
Mild	1 (0.3)	2 (0.6)	0.56
Moderate	3 (0.9)	7 (2.1)	0.20
Severe	293 (88.8)	287 (87.0)	0.47
Mitral regurgitation, n (%)			
None/Trace	172 (52.1)	0 (0)	< 0.001
Mild	158 (47.9)	0 (0)	< 0.001
Moderate/Moderately-Severe	0 (0)	330 (100)	< 0.001
Tricuspid regurgitation			
None	217 (65.8)	216 (65.5)	0.94
Mild	69 (20.9)	66 (20.0)	0.77
Moderate	34 (10.3)	37 (11.2)	0.71
Severe	10 (3.0)	11 (3.3)	0.82

Abbreviations: LVEF = left ventricular ejection fraction

**Table 4:** Propensity-Matched Operative Characteristics

	NoMR	MR	*p*-value
Re-operation, n (%)	105 (31.8)	96 (29.1)	0.45
Minimally invasive approach, n (%)	17 (5.2)	15 (4.5)	0.72
CPB time, minutes (mean ± SD)	89.5 ± 26.6	92.5 ± 33.2	0.19
XCL time, minutes (mean ± SD)	61.9 ± 18.2	63.3 ± 18.8	0.36
Prosthesis type, n (%)			
Biological	299 (90.6)	298 (90.3)	0.90
Mechanical	26 (7.9)	26 (7.9)	1.00
Homograft	5 (1.5)	6 (1.8)	0.76

Abbreviations: CPB = cardiopulmonary bypass, XCL = aortic cross-clamp

Propensity-matched postoperative complications and perioperative mortality are shown in Fig. 1B. There was no difference in 30-day mortality between groups (3.6% MR vs. 3.3% NoMR, *p* = 0.83). Additionally, there were no differences in postoperative complication rates or post-operative length of stay [8 (IQR 6–11) days MR vs. 8 (IQR 6–12) days NoMR, *p* = 0.47). Kaplan-Meier survival analysis of the propensity-matched cohort (Fig. 1C) showed significantly worse medium and long-term survival in Group MR (log rank *p* = 0.02). At 1-year follow-up, mean MR severity was significantly worse in Group MR, although both groups fell in the trace-to-mild range (1.1 ± 0.7 MR vs. 0.8 ± 0.7 NoMR, *p* = 0.03).

## Discussion

Concomitant fMR in patients presenting for surgical AVR remains a challenging clinical problem. Although the predominant opinion is that fMR should improve following correction of aortic valve pathology, it is not clear that postoperative relief of left ventricular pressure-volume overload is sufficient to cause significant regression of moderate fMR, which may subsequently limit functional status and postoperative survival. While it seems that mild preoperative fMR will regress following isolated AVR, studies have shown that many patients with moderate preoperative fMR still demonstrate a clinically significant level of postoperative regurgitation on follow-up [6–23]. Given the lack of well-defined treatment guidelines for these patients and small sample sizes used in prior studies, further investigation of the late effects of isolated AVR on moderate fMR is required.

The current study is, to the best of our knowledge, the largest single-center experience and the only propensity-matched analysis in this patient population. We have demonstrated that unaddressed moderate to moderate-severe (2–3+) fMR at the time of isolated AVR leads to equivalent perioperative survival but worsened medium- and long-term survival compared to patients with no or mild preoperative fMR. We did not detect a difference in postoperative complication rates or postoperative length of stay between groups. One-year echocardiographic follow-up revealed that patients with 2–3+ preoperative fMR had slightly but significantly worse residual MR compared to patients with mild or no preoperative fMR. While the post-AVR left ventricular reverse remodeling may improve fMR severity to some degree, our findings show that preoperative 2–3+ fMR in isolated AVR patients presages poorer late survival.

Review of our overall cohort analysis showed that patients with 2–3+ fMR are sicker than patients with no or mild fMR. Patients in Group MR were significantly older with worse renal and cardiac function, as evidenced by higher rates of congestive heart failure and lower ejection fractions. Group MR patients also had more severe TR, suggesting higher degrees of pulmonary hypertension and right ventricular dysfunction. Several prior studies addressing this population demonstrated similar findings [9, 11, 14]. Although it is possible that the worse prognosis of Group MR patients in the overall analysis is a direct effect of a greater degree of fMR, it is more likely that these patients simply have more advanced cardiovascular disease resulting in worsened overall survival. While our propensity-matched analysis allowed us to control for some of these baseline differences to more specifically evaluate the effect of preoperative fMR, it is worth noting that patients presenting with 2–3+ fMR in the setting of aortic valve disease generally have more advanced cardiac disease than their counterparts with no or mild fMR, and should be treated accordingly.

While many studies have demonstrated that severe MR should be repaired during concomitant AVR, controversy remains concerning the correct management of moderate and moderate-severe MR [2–4, 19–24]. Late survival in prior studies comparing patients with 2–3+ preoperative MR to patients with no or mild MR has varied. Given that the question at hand is whether or not isolated AVR is sufficient treatment for these patients, those studies that found a survival difference generally concluded that mitral intervention should be considered in patients with moderate or greater preoperative MR [6, 7, 13, 19–23]. However, several other studies, including the largest single-center study from the Mayo Clinic (*n* = 190), found no difference in survival between the two groups, suggesting that mitral intervention may be unnecessary [8, 11]. Nonetheless, large baseline demographic differences among groups are present in these studies, fundamentally confounding interpretation of the data. Given the clinical equipoise in the conclusions of prior studies and the potential that moderate fMR may simply be an indicator of more advanced disease rather than a causative entity leading to poorer late survival, a propensity-matched analysis was essential to control for key baseline differences and remove confounding comorbidities. After propensity-matching, our data suggest that a lower bar for mitral intervention in these patients may be warranted. Further study into specific patient subgroups may be necessary to clarify the tradeoff between additional perioperative risk and late mortality with combined aortic and mitral surgery.

Our data suggest that patients with moderate, concomitant fMR undergoing AVR would benefit from mitral intervention, although the logical follow-up question to that observation is to ask whether mitral valve repair or replacement would confer the lowest perioperative mortality and the highest late survival in this population. A Cardiothoracic Surgery Trials Network study from Acker and colleagues evaluated the performance of mitral valve repair vs. replacement for patients with ischemic MR not undergoing AVR and found no difference in perioperative or 1-year survival, but the recurrence rate of moderate or severe MR was higher in the repair group [25]. Although not directly applicable to patients with combined aortic and mitral valve disease, follow-up of this randomized trial could help to predict which mitral intervention confers a greater survival benefit to patients.

Few studies exist that directly address AVR/mitral repair vs. AVR/mitral replacement in the fMR subgroup. In 2003, the Cleveland Clinic group published a study addressing all patients who underwent combined AVR with either mitral valve repair or replacement for any reason, not solely for fMR [26]. While there was no difference in perioperative survival between mitral repair and replacement, they found that concomitant mitral repair with AVR resulted in significantly improved survival at 5, 10, and 15-year follow-up compared to mitral valve replacement with AVR [26]. However, only 7% of the total population had fMR in the Cleveland Clinic series, which again highlights the need for a large randomized trial evaluating the appropriate therapy for patients with aortic valve disease and significant concomitant fMR [26].

There are several limitations to our study. It is a retrospective, single-center study that reflects the treatment biases of our clinical team. We have attempted to limit preoperative baseline differences by propensity matching, but it is possible that our propensity-matched groups do not accurately reflect the true population based on unmeasured covariates. Follow-up survival information did not include the cause of late death, so all comparisons of mortality reflect all-cause mortality and not death from cardiovascular causes. Finally, formal quantification of MR using more advanced echocardiographic parameters, such PISA derived metrics, will be necessary in future studies; these measurements are now a standard part of our echocardiographic analysis, but were not available for the majority of this patient dataset during our study period.

## Conclusions

In conclusion, patients undergoing isolated AVR with concomitant moderate and moderate-severe preoperative fMR have worse medium- and long-term survival, and continue to have elevated MR severity at 1 year, compared to patients with no or mild preoperative fMR. These results indicate the need for mitral valve intervention at the time of AVR in patients with moderate or greater fMR. Given our results and those from prior studies, a randomized trial is needed to definitively clarify the optimal treatment strategy for this population, and to determine whether mitral valve repair or replacement at the time of AVR for moderate or greater preoperative fMR is superior.

## Abbreviations

AI: aortic insufficiency
AS: aortic stenosis
AVR: aortic valve replacement
fMR: functional mitral regurgitation
TR: tricuspid regurgitation
